# Patients without records and records without patients: review of patient records in primary care and implications for surveillance of antibiotic prescribing in rural China

**DOI:** 10.1186/s12913-020-05308-0

**Published:** 2020-06-22

**Authors:** Rachel Kwiatkowska, Xingrong Shen, Manman Lu, Jing Cheng, Matthew Hickman, Helen Lambert, Debin Wang, Isabel Oliver

**Affiliations:** 1 NIHR Health Protection Research Unit (HPRU) in Evaluation of Interventions, Bristol Medical School, Population Health Sciences, University of Bristol, Oakfield House, Oakfield Grove, Bristol, BS8 2BN UK; 2 Field Service, National Infection Service, Public Health England, 3rd floor, 2 Rivergate, Bristol, BS1 6EH UK; 3 School of Health Service Management, Anhui Medical University, 81 Meishan Road, Hefei, 230032 China; 4 Population Health Sciences, Bristol Medical School, Bristol Medical School, Canynge Hall, 39 Whatley Road, Bristol, BS8 2PS UK

**Keywords:** Antimicrobial resistance, Antibiotics, Mixed methods, China, Medical records, Outpatients

## Abstract

**Background:**

We need to monitor patterns of antibiotic prescribing in order to develop and evaluate antibiotic stewardship interventions in rural China. As part of a multidisciplinary study of antibiotic use in Anhui Province we assessed the validity of electronic records (e-records) as a source of surveillance data.

**Methods:**

One township healthcare centre and one village clinic were selected in each of three different counties. Patients with symptoms of Upper Respiratory Tract Infection (URTI), exacerbation of Chronic Obstructive Pulmonary Disease (COPD) or Urinary Tract Infection (UTI) were recruited consecutively. Researchers observed and documented clinic consultations and interviewed each of the study participants. E-records were compared to clinic observations and patient interviews.

**Results:**

A total of 1030 patients were observed in clinic. Antibiotics were prescribed in 917 (89%) of consultations. E-records were created only for individuals with health insurance, with considerable between-site variation in completeness (0 to 98.7% of clinic consultations) and in the timing of documentation (within-consultation up to weeks afterwards). E-record accuracy was better in relation to antibiotics (82.8% of e-records accurately recorded what was prescribed in clinic) than for diagnosis and symptoms (45.0 and 1.1% accuracy). Only 31 participants (3.0%) presented with UTI symptoms.

**Conclusions:**

We have confirmed very high rates of outpatient antibiotic prescribing in rural Anhui province. E-records could provide useful information to inform stewardship interventions, however they may be inaccurate and/or biased. Public Health authorities should focus on improving technical infrastructure and record-keeping culture in outpatient settings. Further research is needed into community treatment of UTIs.

## Introduction

Antimicrobial resistance (AMR) is recognised as one of the greatest public health challenges of our time, predicted to cause an additional 10 million deaths per year by the year 2050 if current global trends prevail [1]. Action to curb antimicrobial resistance, particularly the prudent use of antibiotics (antibiotic stewardship), is essential to maintain the effectiveness of these drugs which underpin modern medical practice.

A detailed understanding of trends and patterns in antibiotic use is essential to identify factors driving inappropriate use and to target interventions to promote antibiotic stewardship. It is important to consider antibiotic use not only in relation to clinical factors, but also human behaviours (both in patients and health professionals) and social and environmental context.

In China, rates of AMR and antibiotic use are known to be very high, resulting in national policy and legislation to promote better stewardship [2]. The National Essential Medicines System (NEMS) scheme, introduced in 2009, allows community hospitals and healthcare centres obtain medications from a provincial supplier to sell to patients at cost price [3]. Medical insurance policies such as the New Rural Cooperative Medical Scheme (NRCMS) cover the costs of medications listed under the NEMS, though physicians may still prescribe ‘non-essential’ medicines where they have procured these from alternative sources and sell them directly to the patient at a mark-up price [4, 5]. The NEMS also implemented protocols and guidelines for the appropriate use of medicines, and the Chinese government launched a campaign in 2011 to reduce inappropriate use of antibiotics in hospitals [2, 6, 7]. These measures have had some effect, although not uniformly across all provinces, nor to the levels recommended by the World Health Organization (WHO) [8]. Restrictive interventions to disincentivise physicians from prescribing antibiotics, particularly intravenous (IV) preparations, have also had limited effect on antibiotic consumption [9].

National surveillance of AMR and antibiotic use draws data from tertiary hospitals and excludes primary care and community hospitals [10–12]. Little is known about the burden of resistance or levels of antibiotic use in rural areas where community healthcare is the mainstay, however several studies suggest liberal and inappropriate use of antibiotics in these settings, both on prescription and over the counter [6, 13, 14]. Although clinical records exist in rural areas (at village and township level) and aggregated data could potentially be accessed for analysis, there is no literature on what is recorded in rural healthcare records, nor whether the data are accurate or complete enough to inform public health action. It is therefore important to identify whether routine clinical records can be used to monitor antimicrobial use and what issues limit the interpretation and generalisability of the data.

## Methods

### Aim and objectives

This research was conducted as part of a large interdisciplinary study investigating levels of antibiotic prescribing and the burden of AMR in rural areas in China [15]. In this component of the study we aimed to assess the feasibility of using routine clinical data to inform antimicrobial stewardship in rural outpatient settings in Anhui province.

Our objectives were to:

1. Describe patient record systems in rural outpatient settings in Anhui province
2. Assess completeness and accuracy of electronic clinical records
3. Assess feasibility of monitoring antibiotic prescribing, clinical indication and microbiological investigations using electronic clinical records.

### Study setting and population

The study was conducted in Anhui Province, central China. Anhui has a population of 62.6 million of whom 46.7% reside in rural areas; the social and cultural profile of this province is considered representative of over 80% of the Chinese population, and per capita GDP ranks 12th among China’s 23 provinces [16].

In Anhui province, as elsewhere in China, there exists a three-tier health system. Patients are free to seek care at any type of healthcare facility although medical insurance systems incentivise the use of first-tier care through greater reimbursement ratios. Accordingly, around two thirds of initial consultations occur in village or community clinics, and township or community health centres receive a slightly greater proportion (17%) of initial consultations than (urban) county-level hospitals (14%). Anhui contains a total of 1095 hospitals, 1882 community and 1367 township health centres, and 15,331 village clinics [17].

Data were collected from one township health centre and one village clinic in each of three different regions within Anhui province. The pilot site is in a county to the north of Anhui’s capital city Hefei and contains a total of 234 healthcare facilities, including 20 township health service centres and 185 village clinics. Site 1 is in a county south of Hefei containing 368 healthcare facilities including 17 township hospitals and 209 village clinics. Site 2 is situated north west of Hefei: this county contains 267 healthcare facilities including 30 township clinics and 104 village clinics.

### Inclusion/ exclusion criteria

Males and females aged 18 years or older were recruited to the study if a) able to consent to microbiological sampling, survey and interview, b) identified by the physician as having one or more of: urinary tract infection; exacerbation of Chronic Obstructive Pulmonary Disorder (COPD); respiratory tract infection; or sore throat and c) presenting in the outpatient setting for the first time within the study period.

### Data sources

In the preliminary phase of the study, field reports provided context and helped inform sampling strategies. Field researchers interviewed directors of two township health centres; four doctors working in Internal Medicine; one surgical doctor; one public health professional responsible for managing regional village clinics; and a selection of village doctors and pharmacists.

Recruitment proformas were used to collect data from patients recruited to the study, including basic demographic and clinical information. Prior to departure from the clinic, study participants completed a more comprehensive exit survey administered by a researcher.

Semi-structured observations were carried out in all the clinics and health centres involved in the study. Researchers filled out worksheets to describe clinic layout and operational procedures for patient triage, consultation, investigation and recall. The worksheet also prompted the researcher to collect details as observed during or immediately after individual consultations. During the clinic consultation, researchers completed a proforma which detailed presenting symptoms (as described by the patient); physician diagnosis (as explained to the patient) and the physician’s approach to recording clinical details (paper or electronic record; completed within or after consultation).

Methods for the review of clinical records were informed by the preliminary field reports (see results section). Data collected during study recruitment and clinic observations were used to assess the completeness and accuracy of routine electronic patient records at each of the study sites. Researchers interrogated electronic records and completed a data collection form relating to variables of interest.

Table 1 outlines variables included in the review of e-records and sources of data for each.

**Table 1:** Data sources and variables used in a review of clinical records for monitoring antimicrobial resistance and prescribing in rural Anhui province

	Data source			
Variable	Recruitment *(Patient)*	Observations *(Physician)*	Exit surveys *(Patient)*	Electronic patient records
Unique study ID	+	+	+	
Date of consultation	**+**		+	+
Location of consultation	+		+	+
Attending physician			+	+
Name	+			+
Date of Birth	+			+
Sex	+			+
Insurance status/ type	+			
Presenting complaint	+		+	
Duration of illness			+	
Symptom(s)	+	+	+	+
Physician diagnosis	+	+	+	+
Investigation(s)	+	+		+
Treatment(s)	+			+
Antibiotic prescribed	+	+	+	+
Antibiotic name(s)	+	+		+
Antibiotic dose(s)	+	+		+
Antibiotic route(s) of administration (IV/IM/PO)	+	+	+	+
Record- keeping practices: (in or post- consultation)		+		
Use of insurance ID (patient or family member)		+		

With the exception of field reports, data from all of the above sources were entered into a central database on a secure Structured Query Language (SQL) server. Each study participant was assigned a unique identifier (ID) which allowed data from different strands of the study to be linked at the patient level.

### Data collection

Study recruitment took place in three phases: 03/05/2017–02/07/2017 (pilot site); 20/10/2017–08/02/2018 (site 1); and 01/03/2018–30/06/2018 (site 2). Patients attending the outpatient settings during clinic hours (8 am-4 pm or 9 am-6 pm) who met the inclusion criteria were recruited consecutively.

Following recruitment, participants were accompanied through their outpatient journey, during which the researcher completed a recruitment proforma, clinic observation worksheet and exit survey.

Two weeks following completion of study recruitment, researchers returned to each study site to search for electronic records (e-records) relating to the consultations that had been observed. Information from e-records was then compared with data from clinic observations and recruitment proformas: researchers documented whether there was agreement between e-record and observations using a template designed for this purpose (see **Appendix)**. At each site, three researchers inputted the data from e-records to the study database, after which a single researcher reviewed all data entries to ensure consistency of approach in coding and interpretation of medical terms.

Data collection forms and templates were translated from English and modified to contextually- specific needs prior to their use; researchers recorded interviews, observations, surveys and e-record reviews in Mandarin.

### Analysis

Field reports were translated into English and analysed by two researchers in the UK. Any information relating to the seeking, provision, funding or recording of healthcare was extracted from the field reports.

Data for the record review were extracted from the central study database into Microsoft Excel files for de-duplication and cleaning. Variable names were translated from Mandarin into English and each downloaded dataset was accompanied by a data dictionary defining codified items.

We assessed completeness and accuracy of e-records. Completion was defined as the proportion of observed consultations with an accompanying e-record. Accuracy was defined as the proportion of e-records which exactly documented what was observed in clinic, for the following variables:

- symptom(s) suggestive of Urinary Tract Infection (UTI), exacerbation of Chronic Obstructive Pulmonary Disorder (COPD), Respiratory Tract Infection (RTI), or sore throat;
- physician diagnosis;
- antibiotic name(s);
- antibiotic dose(s) and
- antibiotic route of administration (intravenous (IV)/ intramuscular (IM)/ oral (PO)).

Symptoms of interest were pre-specified, based on expert knowledge of a UK primary care physician in conjunction with members of the China research team who were familiar with the colloquial terms for infective symptoms (see **Appendix**).

Diagnoses were assigned at the discretion of the attending physician.

For each variable, we calculated the proportion of e-records with an accurate entry (ie matching what was observed in clinic); an inaccurate entry (different from what was observed in clinic); and a missing entry.

Analyses were carried out using Microsoft Excel and R version 3.5.1 [18].

### Patient and public involvement

Patients and the public were not involved in the design of this research study.

## Results

### Clinical record keeping

Field reports described paper records that take the form of log books (a list of attending patients plus basic demographic information), clinical records (comprehensive documentation of demographic and clinical information), and written prescriptions. These tend to be used in village clinics, but not in township clinics or health centres. On this basis, it was decided that e-records should be assessed against direct clinic observations rather than relying on paper clinical records.

E-records are almost exclusively used in township clinics and health centres to log clinical activity for reimbursement from state medical insurance. For this reason, they are created using a patient’s medical insurance number, and a clinical diagnosis which is not always communicated to the patient in clinic. In situations where the patient does not have an insurance ID, or where they have exceeded the individual annual reimbursement allowance for state-funded consultations or treatments, doctors may create (an) e-record(s) using the ID of one or more family members to cover costs.

In village clinics, e-records are less frequently used: low levels of computer literacy and slow computers were cited as inhibitory factors.

Record-keeping practices vary between physicians and from site to site. In village clinics, e-records can be created within the consultation (site 1), some days/ weeks afterwards (pilot site), or once a year when paper records are transposed electronically during prescription audits (site 2). Conversely, in township health centres physicians are obliged to register details of the consultation at the point of care, quoting a medical insurance ID in order that treatments are paid for.

We witnessed treatment seeking in commercial pharmacies, where antibiotics could be purchased over the counter. Pharmacy consultations and transactions are not routinely documented on paper or electronically.

### Symptoms, diagnoses and prescriptions

A total of 1030 individuals were recruited to the study and observed in clinic at the pilot site (*n* = 160); site 1 (*n* = 525) and site 2 (*n* = 345). The majority (87.4%) of study participants had medical insurance under the New Rural Cooperative Medical Scheme (NRCMS). There was no significant variation in the proportion of individuals under NRCMS by study site (Pearson’s Chi-squared test *p* = 0.49).

Most study participants presented with symptoms of respiratory tract infection (RTI) (902/ 1030; 87.6%); throat symptoms were also reported for 760/ 1030 (73.8%) individuals. Only 31 individuals (3.0%) reported symptoms suggestive of urinary tract infection (UTI).

Physicians communicated diagnoses in only 653/ 1030 (63.4%) of consultations. Of the diagnoses verbalised in clinic, Upper Respiratory Tract Infection (URTI) was the most common (*n* = 349; 53.4%) followed by bronchitis (*n* = 100; 15.3%) and pharyngitis (*n* = 65; 10.0%). Only 16 (2.5%) individuals were given a diagnosis of UTI in clinic.

Antibiotics were prescribed in 916 (88.9%) of consultations; of these prescriptions 473 (51.6%) were for intravenous (IV) antibiotics and 384 (41.9%) were for more than one antibiotic. The most commonly prescribed combination of antibiotics was levofloxacin for injection with ceftriaxone for injection (*n* = 32; 8.33% of combination prescriptions) and amoxicillin capsules with levofloxacin tablets (*n* = 26; 6.77% of combination prescriptions).

### Completeness of electronic records

**Fig. 1:**
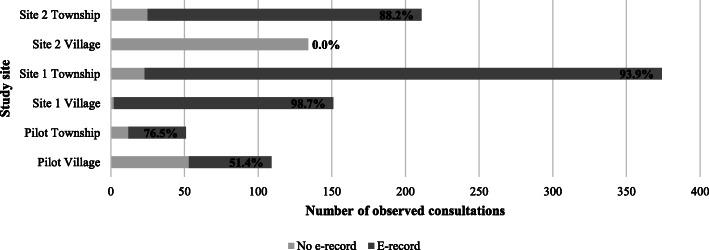
Number (%) of observed consultations with an e-record, by study site

Of the 1030 individuals who were observed in clinic, e-records were identified for 781 (75.7%); the remainder were either non-existent or irretrievable using patient identifiers (name and date of birth). Figure 1 illustrates the distribution of e-records by study site: e-record completion ranged from 0% in site 2 village clinic to 98.7% in site 1 village clinic.

### Accuracy of electronic records

A total of 2637 discrete RTI/ throat/ UTI symptoms (see **Appendix** for symptoms list) were reported by patients during clinic. Only 28 (1.1%) of symptoms recorded in clinic observation worksheets were documented in the corresponding e-record.

Eight percent of symptoms found in e-records had not been recorded during the clinic consultation (Table 2).

**Table 2:** summary of symptoms reported in clinic, in e-record and in both clinic and e-record

Symptom	observed	e-record	accurate e-record (% of observed)	inaccurate* e-record (% of e-records)
blocked nose	232	13	2 (0.9%)	11 (84.6%)
runny nose	199	0	0	0
snotty nose	39	0	0	0
dry cough	124	118	10 (8.1%)	108 (91.5%)
cough green sputum	251	2	1 (0.4%)	1 (50%)
cough clear sputum	41	0	0	0
cough white sputum	360	22	0	22 (100%)
dry throat	146	1	1 (0.7%)	0
sore throat	450	0	0	0
itchy throat	128	0	0	0
burning throat	21	1	0	1 (100%)
hoarse voice	10	0	0	0
difficulty swallowing	5	0	0	0
breathlessness	351	0	0	0
headache	139	13	2 (1.4%)	11 (84.6%)
body pain	7	0	0	0
weakness	32	0	0	0
fever	71	55	7 (9.9%)	48 (87.3%)
urinary frequency	10	0	0	0
urinary urgency	4	7	1 (25.0%)	6 (85.7%)
pain on urinating	13	7	4 (30.8%)	3 (42.9%)
urinary incontinence	2	0	0	0
blood in urine	0	0	0	0
turbid urine	0	1	0	1 (100%)
lower back pain	2	0	0	0
urinary tract itch	0	0	0	0
‘heavy’ stomach	0	0	0	0
**Totals**	2637	240	28 (1.1%)	212 (8.0%)

*inaccurate defined as a symptom recorded in e-record which was not documented in clinic observation

Physician diagnoses were accurately documented (ie they matched the diagnosis given in clinic) in 355/ 781 (45.5%) of e-records. Antibiotic names were accurately documented in 647/ 781 (82.8%) of e-records and doses were correct in 534/781 (68.4%) of e-records. The route of administration for antibiotics (IV/ IM or oral) was documented on e-records from township health centres (*n* = 636), but not from village clinics.

Table 3 summarises the accuracy of e-records in documenting diagnoses and antibiotic prescriptions.

**Table 3:** number (%) of electronic medical records accurately documenting clinic consultations (*n* = 781)

	diagnosis	antibiotic	dose
**absent in e-record**	203 (26.0%)	116 (14.9%)	114 (14.6%)
**accurate e-record**	355 (45.5%)	647 (82.8%)	534 (68.4%)
**inaccurate e-record**	223 (28.6%)	18 (2.3%)	133 (17.0%)

* IV = intravenous; IM = Intramuscular; PO = oral

**Fig. 2:**
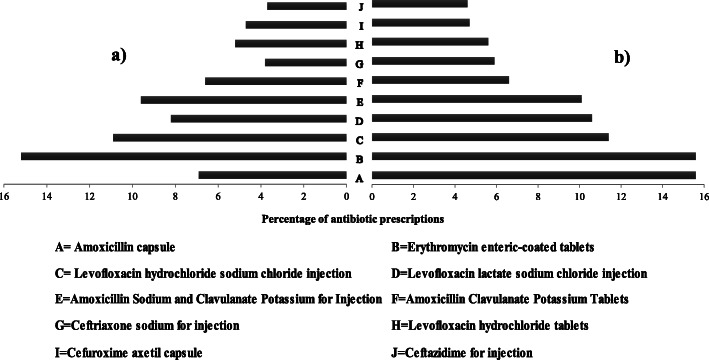
Top 10 antibiotics **a**) recorded in e-record and **b**) prescribed in clinic, as proportion of overall antibiotic prescriptions

On e-record, only 38 different antibiotic types were recorded although in clinic consultations a total of 51 types of antibiotic were prescribed. The 10 most frequently documented antibiotics are shown in Fig. 2. On e- records, amoxicillin capsules constituted a much lower proportion (6.9%) of antibiotic e-prescriptions compared with the proportion that were prescribed on observation (15.6%). Ceftriaxone sodium for injection also featured less on e-records (3.8% of antibiotic e-prescriptions) compared with what was observed (5.9% of prescriptions) as did levofloxacin lactate sodium for injection (8.2% of e-prescriptions versus 10.6% of observed antibiotic prescriptions).

## Discussion

### Main findings

We observed very frequent prescription of antibiotics, a large proportion of which were IV and/ or combination prescriptions in outpatient facilities in rural Anhui province. This highlights the importance of antibiotic stewardship in these settings, and the need to strengthen surveillance to inform and evaluate stewardship interventions. E-records are a useful source of intelligence on antibiotic prescribing, however we have identified significant limitations to their use.

First, record-keeping practices differ between sites and among individual physicians, resulting in variation in the coverage and accuracy of e-records. E-record coverage was particularly varied at the level of village clinics: site 2 lacked the Information Technology (IT) infrastructure to support any routine use of e-records, whereas in site 1 e-records were created for 98.7% of observed consultations.

Second, perverse incentives for creating e-records, designed to claim reimbursement from state insurance for investigations and treatments, leads to inaccurate clinical documentation: only 1.1% of observed symptoms were accurately recorded as compared to 82.8 and 68.4% of antibiotic names and doses, respectively. For consultations in which a diagnosis was verbalised by the physician (653/1030; 63.4%) almost a third of e-records contained an alternative diagnosis, implying that physicians do not routinely use e-records to chart medical histories and/ or inform clinical judgement.

Third, e-records are likely to be systematically biased since they exclude individuals with long-term healthcare needs who have exceeded their insurance credit allowance; those without any medical insurance; and those who are prescribed medications that do not feature in the NEMS or who purchase antibiotics directly from a retail pharmacy [3]. This may explain why 51 different antibiotic names were prescribed in clinic, whilst only 38 were recorded on e-record.

The low number of consultations for UTI is a curious finding and merits further investigation since in many countries, there is a drive to reduce inappropriate use of antibiotics for community-acquired UTI [19].

### Other evidence

The high rates of antibiotic use reported here are not surprising. One study conducted in 23 cities and 16 rural primary care centres in China between 2009 and 2011 found that 52.9% of all outpatients were prescribed antibiotics; another reported that 79.59% of antibiotics prescribed in primary care institutions in Hubei province between May 2011 and November 2013 were administered intravenously [9, 13]. This has been attributed to perverse economic incentives due to remuneration systems that reward physicians on the type and quantity of treatments delivered [5, 20].

Ours is not the only study to identify poor electronic record-keeping culture in rural healthcare facilities in China: others have reported low levels of training and motivation in rural community care, and an ageing workforce which may impact on levels of computer literacy and use of e-records [13, 21].

The observation that physicians in outpatients did not refer to patient clinical records to inform their diagnosis, and in many cases did not provide a diagnosis during a consultation, is echoed by Song et al. who state that treatment guidelines and protocols have yet to be fully adopted in community healthcare centres in China, and that in these settings clinical management tends to focus on symptoms rather than diagnosis [4].

### Strengths and limitations

Our use of direct clinic observations has provided a valid assessment of consultation, prescribing and record-keeping practices in rural healthcare facilities in Anhui province however there are limitations to our approach to reviewing patient records.

Electronic systems were not standardised across all sites as supposed which may have contributed to low concordance between our pre-defined list of symptoms and those retrieved from e-records. The list of symptoms was not validated other than by members of this study team, which may have also contributed to low concordance with e-records.

It was necessary to choose clinics that were within a certain distance from Hefei to allow for samples to be transported to the research laboratory on the day of collection. Results may therefore not represent the most remote parts of Anhui province, nor are they generalisable to other provinces.

## Conclusions

We have confirmed very high rates of antibiotic prescribing for URTI and exacerbation of COPD in outpatient settings in rural Anhui province. Surveillance of antibiotic use is of vital importance to provide clinicians with feedback to inform prescribing practices, and to educate the public to reduce inappropriate demand for antibiotics.

E-records are available for the majority of outpatient consultations and potentially a valuable source of antibiotic surveillance data though issues with coverage and accuracy undermine the validity of these records for surveillance of infections.

Public Health authorities should consider delivering training and IT infrastructure to improve record-keeping culture in rural health facilities. E-record systems should be extended to capture antibiotics provided over the counter, as well as those prescribed under the NEMS. Prescribers should be educated regarding the importance of rational antibiotic use and encouraged to document clinical indications for each prescription.

This study has revealed a surprising lack of presentations with UTI in outpatient clinics: further research is needed to investigate treatment seeking behaviours for urinary infections in rural China.

## Data Availability

The datasets used and/or analysed during the current study are available from the corresponding author on reasonable request.

## Appendix

### Data collection template for review of electronic records

**Table:**

	Patient ID	Date of review	Study site	Date of clinic	Physician name	Observed symptom	Observed diagnosis	Observed antibiotic	Observed dose	e-record exists	e-record symptom	e-record diagnosis	e-record antibiotic	e-record dose
**Code**						0 = absent				0 = no	0 = incomplete			
						1 = present				1 = yes	1 = accurate*			
											2 = inaccurate*			
*e-record matches/ differs from observation														

Summary of terms recorded by researchers in review of electronic records.

**Table:**

**Respiratory symptoms**: blocked nose; runny nose; snotty nose; dry cough; cough green sputum; cough clear sputum; cough white sputum; difficulty breathing; headache; body pain; weakness; fever **Throat symptoms**: dry throat; sore throat; itchy throat; burning throat; rough/ hoarse voice; difficulty swallowing; **Urinary symptoms**: urinary frequency; urinary urgency; pain/ difficulty urinating; blood in urine; turbid urine; lower back pain; urinary tract itching; lower stomach feeling heavy.	
**Diagnoses**: bronchitis (acute/ chronic); bronchial infection; bronchiectasis; cold; pharyngitis; Upper Respiratory Tract Infection (URTI); tonsillitis; tracheitis; Urinary Tract Infection (UTI)	
**Top 10 prescribed antibiotics**: amoxicillin capsule; amoxicillin sodium clavulanate potassium tablets; amoxicillin sodium clavulanate potassium injection; ceftazidime for injection; ceftriaxone sodium for injection; cefuroxime axetil capsule; dirithromycin enteric coated tablets; levofloxacin hydrochloride sodium chloride injection; levofloxacin lactate and sodium chloride injection; levofloxacin hydrochloride tablets	

## Abbreviations

AMR: Antimicrobial Resistance
COPD: Chronic Obstructive Pulmonary Disorder
E-record: Electronic patient care record
GDP: Gross Domestic Product
ID: Identifier/ Identification
IM: Intramuscular
IT: Information Technology
IV: Intravenous
NEMS: National Essential Medicines System
NRCMS: New Rural Cooperative Medical Scheme
PO: Per Os (oral)
RTI: Respiratory Tract Infection
SQL: Structured Query Language
URTI: Upper Respiratory Tract Infection
UK: United Kingdom
UTI: Urinary Tract Infection
WHO: World Health Organization
